# Ten Papers that Changed My Practice in Neurocritical Care

**DOI:** 10.5005/jp-journals-10071-23197

**Published:** 2019-06

**Authors:** Srinivas Samavedam

**Affiliations:** Department of Critical Care, Virinchi Hospital, Hyderabad, Telangana, India

**Keywords:** Acute ischemic stroke, Anti epileptic drugs, Intra cerebral Hemorrhage, Neurocritical care, Window period

## Abstract

Neurocritical care is an ever growing specialty with paradigm changes being the norm. Some of the established practices like triple H therapy for SAH and steroids in pyogenic meningitis have seen major changes in published evidence. This article evaluates the evidence published over the last few years which resulted in a change in the authors approach to and practice of neuro intensive care.

**How to cite this article:**

Samavedam S. Ten Papers that Changed My Practice in Neurocritical Care. Indian J Crit Care Med 2019;23(Suppl 2):S165–S168.

Neurocritical care has shown a significant change in paradigms over the last 5 years, be it the window periods for stroke intervention or the role of targeted temperature management. I have put together some of the papers which have brought about these paradigm changes.

## HEMICRANIECTOMY FOR MALIGNANT MCA INFARCTION IN OLDER PATIENTS

Malignant MCA infarction is generally associated with adverse outcomes and high morbidity. While the benefits of a decompressive hemicraniectomy were clearly known in younger patients (< 60 years of age), the role of the intervention in older population was not studied until the destiny investigators asked the question.^1^

Juttler et al examined whether individuals older than 60 years of age, who developed a malignant MCA infarction, do better with a hemicraniectomy. This was a non blinded RCT carried out in 13 German hospitals between 2009 and 2013. The craniectomy was done within 48 hours of the development of the infarction. The patients who underwent the craniectomy had poorer Rankin scores at 6 months. Survival of these patients however seemed to be higher. The authors concluded that early hemicraniectomy increased survival of older patients with malignant MCA infarction with significant disability.

### How my Practice was Influenced?

This study alerted me to be more practical while recommending hemicraniectomy for malignant MCA infarcts in older patients. I started discussing the need for rehabilitation more while recommending the procedure.

## MONITORING INTRA CRANIAL PRESSURE (ICP) IN TBI

Traumatic brain injury is probably the first area of intensive care where the principles of neuromonitoring initially emerged. Monitoring ICP was considered as a standard of care for TBI. Decisions of surgery were driven by the guidelines which focused on the ICP. Therapy was always tailored along those lines. However Chestnut et al got everyone thinking about this approach after the publication of the BEST: TRIP study.^2^

The clinical question which Chestnut et al. attempted to answer was whether ICP monitoring improves mortality and neurological function among patients with severe TBI. ICP was monitored using a parenchymal probe for a mean duration of 3.6 days. Critical value of ICP was accepted as 20 mm Hg. The control group was monitored using imaging techniques.

A cohort of more than 300 patients was included over 3 years in six Bolivian and Ecuadorian centers. Patients who were monitored by imaging received more hypertonic saline and more hyperventilation. Mortality did not differ in both the groups. Surgery rates also did not differ between both the groups.

The authors concluded that ICP monitoring guided therapy did not confer much benefit among patients with TBI.

### How my Practice was Influenced?

This study alerted me to the fact that invasive ICP monitoring is probably not the only way to improve outcomes among patients with TBI. However, some form of estimation of the pathological process with imaging atleast is needed to deliver acceptable care to the patients.

## THERAPEUTIC HYPOTHERMIA FOR RAISED ICP ASSOCIATED WITH TBI

Therapeutic hypothermia is an integral part of “neuroprotective” measures across a spectrum of neurological emergencies. TBI being the foremost amongst these is obviously a target. Reduction in mortality and long-term disability are the main parameters to judge the efficacy of such measures including therapeutic hypothermia.

Andrews et al attempted to put this hypothesis to test through the Eurotherm 3235 trial ^3^. The study sought to evaluate the effect of hypothermia (32–35° C) on 6 month disability and survival. Forty seven centers across 18 countries enrolled close to 380 patients. Patients with a closed TBI and an ICP > 20 mm Hg were included. Hypothermia was applied for atleast 48 hours and continued till ICP stabilized. The non hypothermia group appeared to do better on the Glasgow outcome scores at 6 months. The number needed to harm was reported to be 10. Six month mortality seemed to be higher in the hypothermia group. Serious adverse events were more common in the hypothermia group. However, the study was criticized for changing the age limits and the time duration of injury after the recruitment started.

### How my Practice was Influenced?

This study made me to rethink about the pathophysiology of TBI vis-à-vis other forms of neurological injury. Loss of autoregulation seemed to be a differentiating factor. I now use of hypothermia more selectively and discretely after this study.

## AGGRESSIVE LOWERING OF BLOOD PRESSURE FOLLOWING ACUTE INTRACEREBRAL HEMORRHAGE

Intracerebral hemorrhage is a catastrophic event, more often seen in hypertensive individuals. It seemed logical that lowering of blood pressure to “normal” levels aggressively could prevent the recurrence or expansion of the bleed.

This logic was put to test by the ATACH 2 investigators.^4^ Qureshi et al evaluated the effect of lowering systolic blood pressure to 139–110 mm Hg among hypertensive patients who have had an intracranial bleed. The study had a follow-up period of 3 months and was run in multiple countries over 4 years. Infratentorial and intraventricular bleeds were excluded. Treatment was initiated when systolic blood pressure exceeded 180 mm Hg. The target was 110–139 mm Hg (intensive treatment) vs 140–179 mm Hg (standard treatment). Nicardipine and IV Labetalol were the two drugs used to achieve control of blood pressure.

There was no significant difference in long term disability and outcome between those who had aggressive lowering of blood pressure and those who did not. Significant adverse events were more frequent in those whose blood pressure was aggressively lowered. Moreover, it was very difficult to achieve the targets of blood pressure lowering within 2 hours of initiation of therapy. The authors concluded that it is not beneficial to aggressively lower the blood pressure in hypertensive intracerebral bleeds.

### How my Practice was Influenced?

The enthusiasm in lowering blood pressure in hypertensive bleeds was tempered after the results of this study. The potential for harm with an aggressive approach towards blood pressure is what essentially changed my practice.

## PLATELETS FOR INTRACRANIAL HEMORRHAGE SECONDARY TO ANTIPLATELET THERAPY

Antiplatelet therapy is fairly common amongst the general population for a variety of clinical reasons. Intracranial bleeds are also becoming more frequent amongst this cohort of patients. It is a popular sentiment amongst medical professionals that platelet transfusion is essential and is beneficial in patients who develop intra cranial hemorrhage while on antiplatelet agents.

Baharoglu et al. in a small RCT examined the safety and benefits of platelet transfusions while treating patients with intracranial hemorrhage due to antiplatelet agents. ^5^

The authors included all patients with non traumatic supra tentorial intra cerebral hemorrhage while on atleast a week of antiplatelet therapy. The patients all had a GCS between 8 and 15. Aspirin, clopidogrel and dipyridamole were the antiplatelet agents which were on board when the bleed happened. Intraventricular bleed category had higher representation in the intervention group at base line. Leucocyte depleted random donor platelets were used (5 for aspirin related and 10 for other drug related bleeds).

The intervention group reported more deaths and disability at 3 months with no difference in the rate of increase of the hematoma. The original size of the hematoma also did not make a difference to the outcome.

The authors felt that platelet transfusions cannot be recommended for the management of antiplatelet therapy related intra cranial hemorrhage.

### How my Practice was Influenced?

This study changed my perspective on the issue of antiplatelets and IC bleeds. My tendency to recommend transfusions to all patients with intracranial bleed if they had antiplatelets in their past medical history made an about turn.

## INFERIOR VENA CAVA DISTENSIBILITY IN SUBARACHNOID HEMORRHAGE

Subarachnoid hemorrhage frequently throws up challenges in terms of hemodynamics and intravascular volume management. The complications triggered by hypovolemia and low perfusion pressures are well known. Conventional hemodynamic monitoring techniques have their own intrinsic problems in execution and actionability. The arrival of sonographic assessment to the bed side raised the possibility of using this modality for management of fluids in patients with SAH.

Morretti et al. compared the distensibility of IVC with thermodilution derived cardiac index and SVV among patients with (Fisher grade 3/4) SAH.^6^

This study specifically evaluated patients with higher grades of SAH and lower cardiac index. It is a single center observational study done over a one year period. Patients with grade 3/4 SAH who had a cardiac index <2.5 L/min/m^2^ or a CPP <60 mm Hg were included while excluding patients with arrhythmias, ARDS and high EVLW.

Fluid challenge was given with 7 mL/kg of starches which is a questionable step in current practice. Two experienced sonologists performed the IVC assessment at zero PEEP. Measurements of maximum inspiratory diameter and minimum expiratory diameter were measured along with calculation of distensibility index. The parameters so obtained were compared against PICCO derived cardiac index and SVV. The reliability of dIVC in predicting fluid responsiveness was good with a highly satisfactory AUROC. Inter observer variability was also within acceptable limits.

### How my Practice was Influenced?

The paradigm shift from mandatory invasive monitoring for reliability toward equally trustworthy method like ultrasound was reinforced by the findings of this small study.

## THROMBECTOMY FOR STROKE AT 6–16 HOURS WITH SELECTION BY PERFUSION IMAGING

Management of stroke has become an exact science ever since thrombolysis became an accepted intervention. The results of TLT when properly delivered are gratifying. However, the non-applicability of this therapy beyond window period necessitated a search for other modalities.

Albers et al. evaluated the role of mechanical thrombectomy done 6–16 hours after the onset of ischemic stroke related to proximal ICA or MCA.^7^

This was a multicenter RCT which centered around all patients receiving CT perfusion imaging or MRI with diffusion carried out over one year in the US. Patients who presented with occlusion of proximal MCA-M1 or ICA on CT/MR angiography, Infarct volume < 70 mL on imaging, Ischaemic tissue: Infarct volume ratio >= 1.8 and absolute volume of potentially reversible ischaemia (penumbra) >=15 mL were evaluated and included provided intervention could be initiated between 6 hours and 16 hours after the time the patient was known to be well last.

Mechanical thrombectomy was compared to medical therapy which included Aspirin, IV tPA without dual antiplatelet agents and oral anticoagulants. The thrombectomy group had better Rankin scores and were more functionally independent at 90 days. The number needed to treat for a beneficial effect was 4. This study had to be stopped early because the results of the DAWN study forced an unscheduled interim analysis and the proof of benefit was unequivocally shown. Reperfusion rates were also superior with no increase in adverse events.

### How my Practice was Influenced?

The diagnostic and therapeutic nihilism that I tended to have when faced with an ischemic stroke beyond the conventional window period, dissipated somewhat post this publication, reinforced by the DAWN study. This study had broader inclusion criteria than the DAWN study and was not industry sponsored unlike the latter.

## LEVETIRACETAM VS PHENYTOIN FOR STATUS EPILEPTICUS

Status epilepticus is a challenge in the neuro-ICU. Although several well validated guidelines are available, the emergence of Levetiracetam as a convenient antiepileptic drug (AED) with less drug interactions than Phenytoin have made its usage in neuro ICU more common.

Gujjar et al. evaluated the ability of phenytoin and levetiracetam in ability to control seizures in a prospective RCT. ^8^

The study was carried in a single university teaching hospital in Oman. Fifty-two patients with status epilepticus and 63 patients with cluster seizures were included in this study. Levetiracetam at 30mg/kg IV over 30 minutes was compared against phenytoin 20 mg/kg IV over the same time. Benzodiazepines were common to both the groups.

Control of seizures was comparable between the two groups and between the two types of seizures. Functional outcome was better with levetiracetam in the cluster seizure group but not the status epilepticus group. Mortality was also not different between the two groups.

The study concluded that the safety and efficacy of levetiracetam was comparable to phenytojn.

### How my Practice was Influenced?

Levetiracetam being a new drug was always a second choice to phenytoin when I faced a patient with status epilepticus. I had some concerns about the safety of phenytoin when rhythm was unstable or hemodynamics were labile. This subgroup was excluded from the study. However, the study gave me the confidence that levetiracetam is an equal and effective alternative to phenytoin if I did not want to use the latter.

## EARLY MANIPULATION OF ARTERIAL BLOOD PRESSURE IN ACUTE ISCHEMIC STROKE

Manipulation of blood pressure has always been an integral part of Stroke management. While aggressive lowering of blood pressure showed no benefit in hemorrhagic stroke (as discussed earlier), the method of addressing blood pressure in ischemic stroke needed further study.

Nasi et al.^9^ compared three ranges of blood pressure — 140–160 mm Hg (median BP achieved: 153 mm Hg), 161–180 mm Hg (median BP achieved: 163 mm Hg) and 181–200 mm Hg (median BP achieved: 178 mm Hg)—and compared the outcome against each other. Fluids were used to augment blood pressure with esmolol being used to titrate the target blood pressure.

The modified Rankin score did not differ the three groups. The incidence of symptomatic intra cerebral bleed was higher in the highest systolic blood pressure group. Outcome was the best in the group who did not require any manipulation of blood pressure. Adverse incidents were more often identified when noradrenaline was used to manipulate blood pressure.

### How my Practice was Influenced?

This study laid out the principles of not generating numbers to achieve an outcome. It also established the role of blood pressure manipulation only when the SBP crossed 180. This study gave a target (161–180) to be maintained for acute ischemic strokes.

## THROMBOLYSIS GUIDED BY PERFUSION IMAGING UP TO 9 HOURS AFTER ONSET OF STROKE

Thrombolysis is still the more easily available therapeutic modality for acute ischemic stroke. At the same time the number of patients who reach the care facilities within the accepted window period of 4.5 hours is less. Extending this therapy to patients who present later needs to be investigated.

Ma et al. looked at this issue and evaluated whether alteplase administered 4.5–9 hours after stroke onset confers a functional benefit.^10^

All patients who presented 4.5–9 hours post stroke onset underwent CT /MRI perfusion imaging. Those who had hypoperfused but salvageable regions of the brain were randomized to receive alteplase 0.9 mg/kg (max 90 mg) administered as a 10% bolus and 90% infusion over 1 hour or a matching placebo. The intervention group had better Rankin scores at 90 days and significantly better reperfusion at 24 hours.

The authors feel that more studies are needed to identify longer window periods.

### How my practice was influenced?

This study brought the concept of precision medicine and individualized decision making into focus and alerted me to the fact that stroke therapy is not limited to narrow window periods.
